# How social is social resistance?

**DOI:** 10.1042/EBC20250041

**Published:** 2026-09-02

**Authors:** Rosie Randall, Ashleigh S. Griffin

**Affiliations:** Department of Biology, University of Oxford, South Parks Road, OX1 3EL, Oxford, U.K.

**Keywords:** β-lactamase, antibiotic resistance, cooperation, social microbiology

## Abstract

β-lactamases are central to bacterial resistance against β-lactam antibiotics and are often treated as a textbook example of cooperative resistance. The cooperative aspect of their action arises from specific details of their action and location. Firstly, they degrade β-lactam antibiotic molecules, which detoxifies the environment for neighbouring cells as well as self, and secondly, they can act extracellularly, increasing the spatial range of this detoxifying effect and the benefit to neighbouring cells. Non-producer cells are able to take advantage of cross-protection from their cooperative neighbours and cooperator–cheat dynamics can emerge. However, mechanistic and ecological details determine the extent to which β-lactamase evolves as a result of its social action, or from private benefits to the producing cell. In the present review, we highlight recent work that reveals substantial variation in the extent to which β-lactamase-mediated resistance is shared among cells, focusing on subcellular localisation, membrane anchoring, outer-membrane vesicles, and population structure. We argue that β-lactamase spans a continuum from private to cooperative, and that recognising this variation is important both for understanding the evolution of antimicrobial resistance and for predicting treatment outcomes.

## Introduction

Cooperation, whether performed between bacterial cells, meerkats, or humans, is formally described as a behaviour that benefits another individual and has evolved, at least in part, as a result of that benefit [1]. In microbial systems, the majority of cooperative traits involve contribution to a shared resource pool of secreted products, commonly known as ‘public goods’ [2]. Examples include compounds such as iron-chelating siderophores, biofilm matrix compounds, and quorum-sensing signals, all of which have been experimentally demonstrated to be favoured, at least in part, by their beneficial effect on others [3–5]. Public goods production of any kind is vulnerable to exploitation by non-producing cheats, leading to cooperator–cheat competitive dynamics [3]. Social evolution theory provides us with the tools to make predictions about when cheats are likely to be favoured, and these predictions have been tested experimentally in bacterial systems [6–10]. The present review explores the relevance of this field for our understanding of antibiotic resistance.

**Figure 1:**
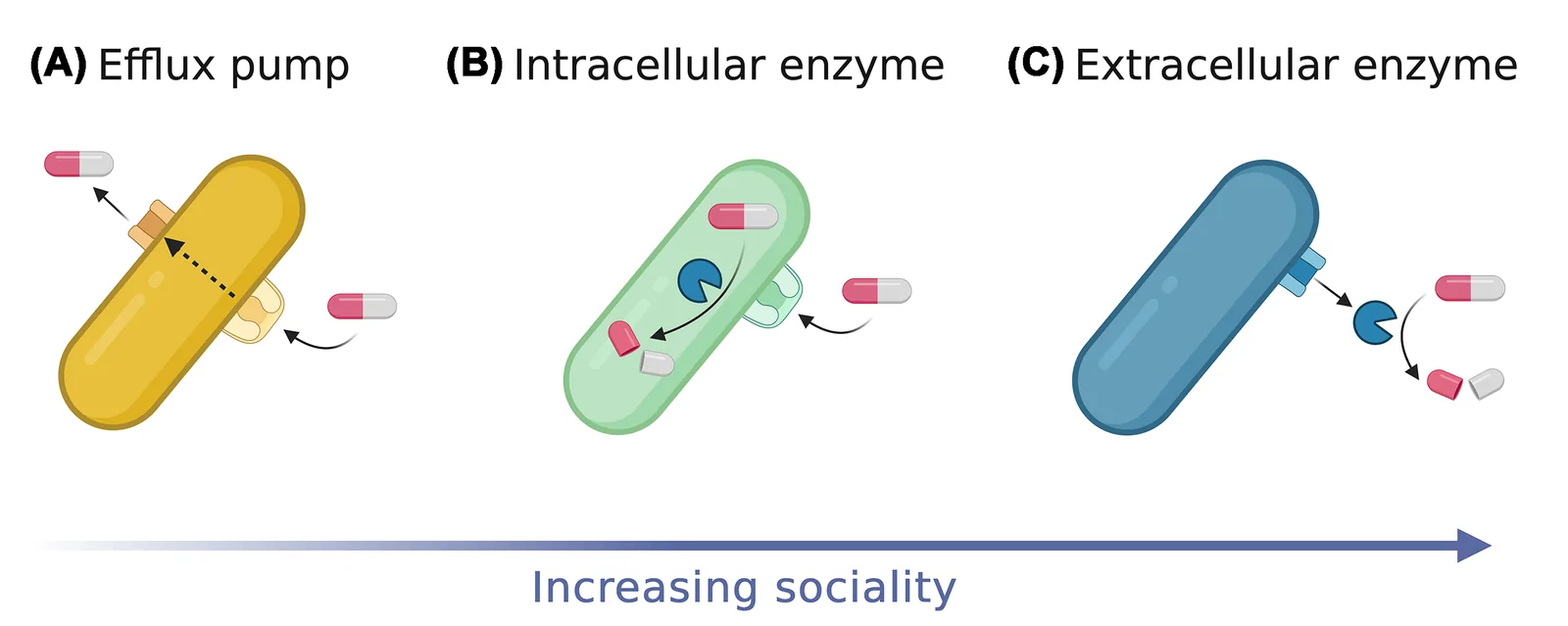
Antibiotic resistance mechanisms categorised by social effects (**A**) Bacterial cells become resistant to antibiotics such as fluoroquinolones by an efflux pump mechanism. In this case, only the focal cell is protected and antibiotic compounds remain intact in the environment. (**B**) Resistance to some antibiotics, such as aminoglycosides, relies on the production of an intracellular enzyme. The focal cell is protected but the concentration of antibiotic is reduced, which potentially benefits neighbouring cells. (**C**). Resistance to β-lactam antibiotics can involve secretion of extracellular enzymes, which can potentially act as public goods and are, therefore, potentially exploitable. [Image created with BioRender.com and used with permission from creators Anna Dewar and Laurie Belcher.]

**Figure 2:**
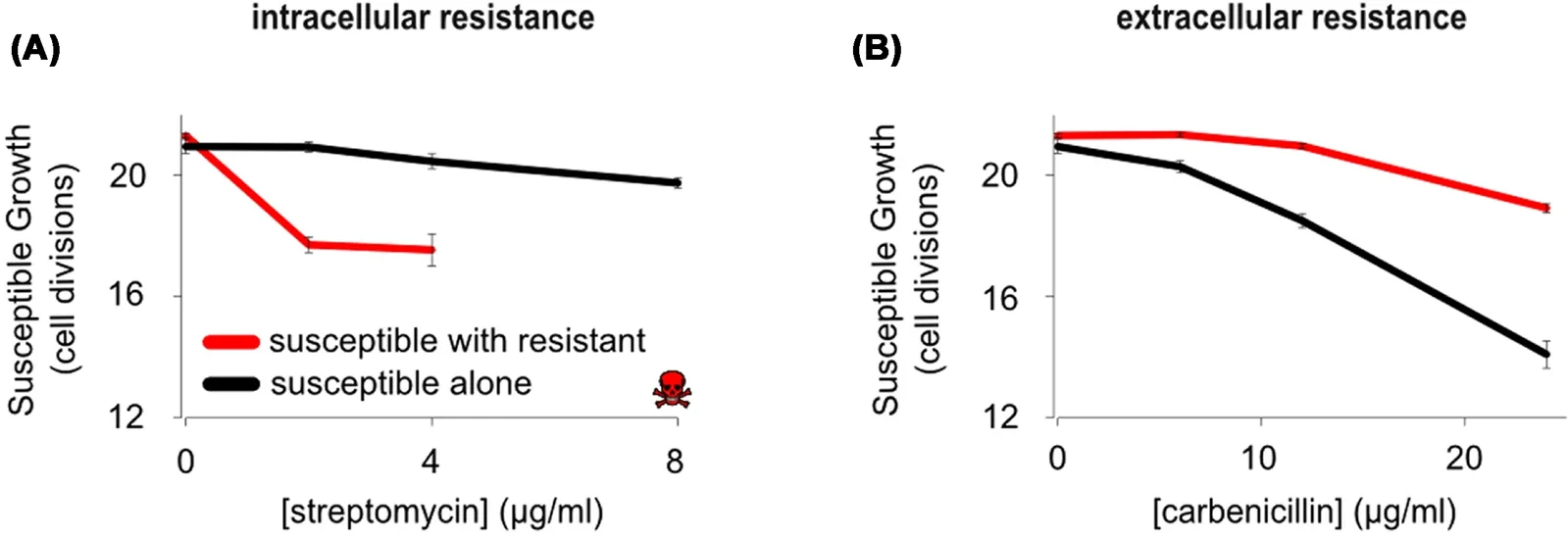
Experimental evidence for cooperative resistance Frost et al*.* competed antibiotic susceptible and resistant strains on solid agar with either streptomycin or carbenicillin. Notably, resistance to streptomycin was via an intracellular mechanism and resistance to carbenicillin was via an extracellular mechanism (β-lactamase production). (**A**) The susceptible strain was outcompeted by the resistant strain at all streptomycin concentrations. (**B**) However, on carbenicillin the resistant strain instead cross-protected the susceptible strain [figure from Frost et al.)].

β-lactamases are enzymes produced by bacterial cells to hydrolyse β-lactam antibiotics and are a major mechanism of antimicrobial resistance in both Gram-negative and Gram-positive bacteria [11,12]. They are often characterised as an example of a public good due to the fact that their action detoxifies the environment for neighbours as well as for the producing cell (Figure 1) [13–16]. This cross-protection effect has been demonstrated experimentally. Frost et al*.* competed antibiotic susceptible and resistant strains of *Pseudomonas aeruginosa* in broth culture and on agar plates (Figure 2) [13]. The susceptible and resistant strains were isogenic; however, the resistant strains were transformed with a plasmid conferring resistance to both streptomycin and carbenicillin. An intracellular resistance mechanism based on production of aminoglycoside adenyltransferase provided resistance to streptomycin and resistance to carbenicillin was via extracellular β-lactamase production. In liquid cultures, Frost et al*.* found no evidence for cooperative resistance for either aminoglycoside adenyltransferase production (intracellular) or β-lactamase production (extracellular). However, evidence was found for cooperative effects of β-lactamase production on agar plates, which were exploited by non-producing cheats at low carbenicillin concentrations. This is consistent with predictions from social evolution theory as spatial structuring is likely to lead to higher relatedness between interacting cells [3,6]. When colonies were mixed with a pipette tip to break up the relatedness structure, cheater cells were once again able to invade.

Although we have evidence to support the action of β-lactamase as a public good from carefully controlled laboratory systems, such as the one described above [13–16], the relevance of social effects in infections is harder to demonstrate. However, there are now clear clinical examples where social protection has been implicated in treatment failure [17]. β-lactamase secreted *in vivo* by amoxicillin-resistant *Haemophilus influenzae* have been shown to protect co-infecting group A *Streptococcus* during therapy, leading to β-lactam treatment failure in a human pharyngotonsillitis case [18]. More broadly, gut microbiome studies and infection models indicate that β-lactamase producers can shield susceptible cells and alter infection outcomes, supporting the idea that cooperative resistance can shape the course of real-world infections [19,20].

One way to address the challenge of identifying social effects in natural populations is to examine signatures of kin selection in the genome [21]. Linksvayer and Wade took a population genetics approach and predicted higher polymorphism in cooperative genes—this prediction arises from the expectation that selection will be less efficient if fitness effects also depend on the success of relatives as well as the individual expressing the allele [22]. Belcher et al*.* examined natural populations of both *P. aeruginosa* and *Bacillus subtilis* and took advantage of variation between different resistance mechanisms and their potential to be social [23,24]. They compared patterns of nucleotide polymorphism and divergence between potentially cooperative resistance genes (β-lactamases and aminoglycoside modifiers) and more private resistance genes (efflux pumps) (Figure 1). Elevated polymorphism and divergence in the β-lactamases and aminoglycoside modifiers are consistent with indirect fitness benefits to relatives playing a more important role in their evolution [22].

Broader genome-wide analyses have reached similar conclusions for cooperative traits more generally. In a natural population of bacteria, genes encoding extracellular products such as secreted enzymes and siderophores show elevated nonsynonymous divergence compared with genes for private traits [22,24]. Estimates of relatedness between interacting individuals are often high (*r* ≈ 0.8), as predicted if kin selection is an important driver [23,24]. Together, these results suggest that cooperative resistance genes, including β-lactamases, fit into a broader pattern in which social interactions and kin selection leave detectable genomic signatures.

A social benefit does not imply that a behaviour does not also bring selfish or private benefits—even if β-lactamase has social effects, these may be trivial compared with the selfish benefits of being resistant. The key question is perhaps not whether β-lactamase production is a social trait but how social is it? To what extent do we expect evolutionary conditions favouring cooperation to impact on patterns of resistance via β-lactamase production? In the following sections, we highlight recent insights into the mechanistic details of β-lactamase production that allow us to make much more nuanced predictions about β-lactamase production. When is it useful to apply social evolution theory to understand its dynamics and when should we expect it to behave like a more private resistance mechanism (Figure 1)?

## Variation in social effects of β-lactamase

The extent to which β-lactamase production has a fitness impact on neighbours is predicted by social evolution theory to depend on factors such as how much enzyme is produced, how fast, and how much area is detoxified around the producing cell [6,25]. These factors are in turn likely to be determined by viscosity, cell density, and the precise details of how β-lactamase is released [9]. Recent experimental work supports the potential for variation in these social effects in *Escherichia coli* [26]. Wang et al. assayed cross-protection across seven clinically relevant β-lactamases in a common genetic background and found differences in how strongly each enzyme rescues non-producing neighbours at sub-inhibitory and inhibitory antibiotic concentrations. When β-lactamases were chromosomally encoded, they were less effective at cross-protection than when encoded on a plasmid, supporting their expectation that a higher copy number of β-lactamase genes results in stronger cross-protection of non-producing cells.

Microfluidic and biofilm studies similarly show that cross-protection depends on ecological context: under certain antibiotics, producer densities, and spatial structures, a minority of β-lactamase-producing cells can shield an entire mixed population, whereas in well-mixed broth the same enzymes confer much weaker benefits to non-producers [16,27,28]. Work on dormancy and persistence further demonstrates that susceptible or persister cells can exploit β-lactamase-mediated detoxification, but only under particular combinations of producer frequency, diffusion, and exposure history [16]. Taken together, these studies reinforce the idea that β-lactamase can function as a public good, while simultaneously pointing to substantial variation in its cooperative potential, both among enzymes and across environments.

### Consequences of plasmid-borne transmission for social effects

Clinically important β-lactamases are frequently carried on mobile genetic elements. In *E. coli*, population genomic surveys indicate that a substantial fraction of β-lactamase genes are plasmid-borne, often on conjugative plasmids, rather than on the chromosome [29,30]. Plasmid carriage can influence β-lactamase expression through increased gene copy number and impose additional fitness costs [31], both of which have the potential to shape the social dynamics of resistance by increasing the metabolic burden of public good production. More work is needed here: these effects are highly context dependent and have not been systematically quantified for most β-lactamases.

Plasmid transmission has been suggested more generally as a mechanism for enforcing cooperative public good production and limiting the ability for cheats to invade [32,33]. Producer cells can ‘infect’ cheats with a cooperative gene transferred on a plasmid. Given the prevalence of β-lactamase production genes on plasmids, it is possible that this mechanism helps to maintain cooperative resistance. Although intuitively appealing, the theoretical and empirical evidence does not support the generality of this as a mechanism for the maintenance of cooperation, including social resistance. There is no evidence that cooperative genes are more likely to be located on plasmids across species [34].

## Mechanistic drivers of variation in social effects of β-lactamase

### Subcellular localisation

**Figure 3:**
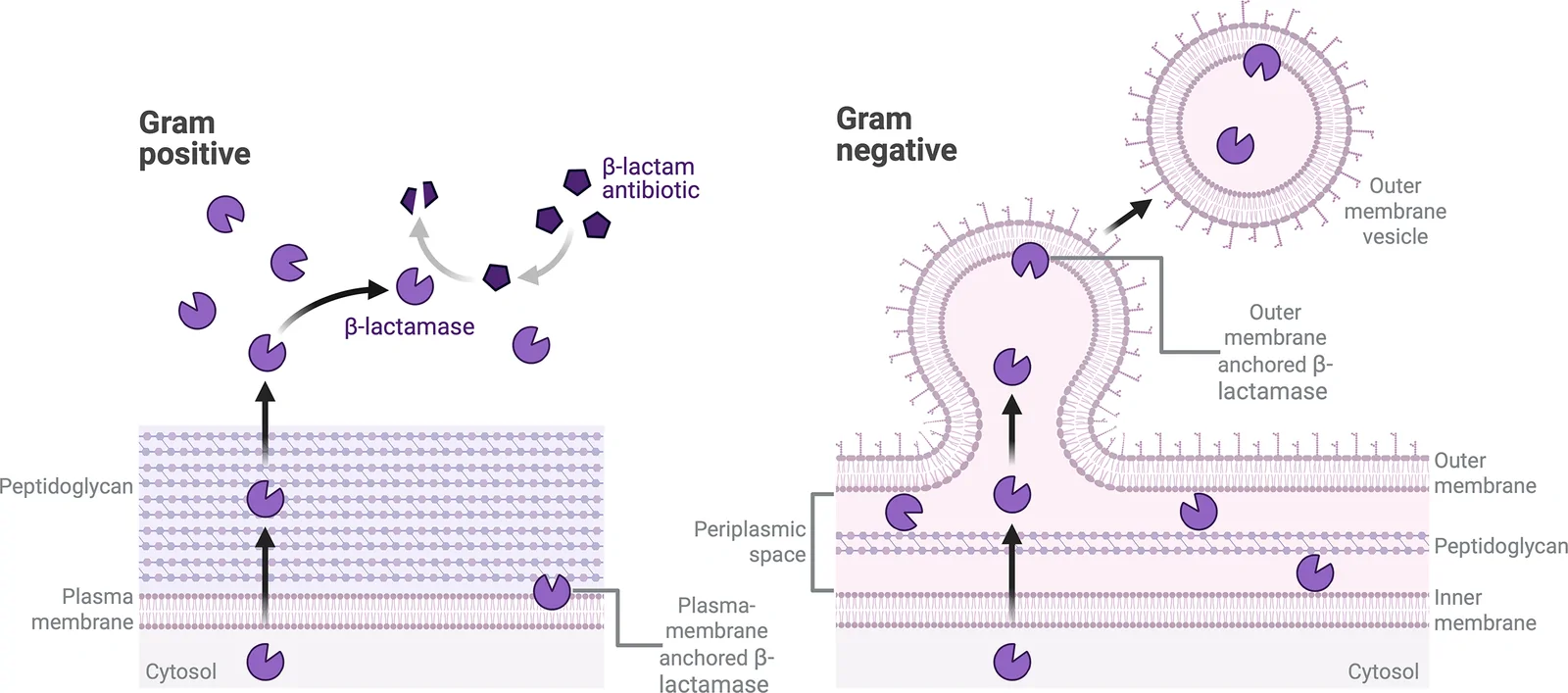
Subcellular localisation of β-lactamase In Gram-positive bacteria (left), β-lactamases are generally secreted straight into the extracellular space. However, they can also be anchored to the plasma membrane. In Gram-negative bacteria (right), β-lactamases are generally located in the periplasmic space. However, they can also be secreted in outer-membrane vesicles, as well as anchored to the outer membrane. [Created with BioRender.com.]

β-lactamases are often characterised as an extracellular mechanism of antibiotic resistance. They are commonly assumed to be secreted into the extracellular space, where they degrade β-lactam antibiotics much like other well-studied public goods such as siderophores. However, evidence that β-lactamases are freely released into the extracellular matrix is limited, and their typical localisation differs between Gram-negative and Gram-positive bacteria (Figure 3) [12]. In Gram-negative bacteria, most β-lactamases are made inside the cell and then guided across the inner membrane by a short targeting sequence at their beginning (an N-terminal signal peptide), so that they end up in the periplasm, where they hydrolyse antibiotics as they diffuse into the cell [35,36]. Because β-lactamases are too large to pass through outer-membrane porins, many remain periplasmic rather than being secreted directly into the external environment [37]. β-lactamases can nevertheless reach the surrounding environment as cargo of outer-membrane vesicles, or via other release mechanisms [38,39], raising a mechanistic and social question: does a β-lactamase confined to the periplasm provide the same protection to neighbours as β-lactamase carried in outer-membrane vesicles or otherwise released extracellularly?

Recent experimental work has begun to disentangle the contributions of periplasmic and extracellular β-lactamases. In *E. coli*, cooperative resistance in coculture correlates most strongly with total β-lactamase activity, including intracellular contributions, rather than with extracellular activity alone, indicating that periplasmic β-lactamase can also drive social effects by lowering β-lactam concentrations outside the cell through diffusion [26]. Extracellular β-lactamase activity still plays a role, however, because high levels of enzyme released into the medium or carried in outer-membrane vesicles can create ‘legacy’ protection in the environment that persists even when producer density falls. This has been experimentally shown by growing β-lactamase-producing bacteria with different antibiotics, extracting the supernatant, and demonstrating that the supernatant can cross-protect non-producing cells [26]. Similar patterns have been observed for other intracellular resistance enzymes, such as chloramphenicol acetyltransferase, where antibiotic inactivation inside resistant cells reduces extracellular drug levels and rescues neighbouring susceptible bacteria [40,41]. These findings imply that the public good in cooperative resistance is best thought of as the local removal of antibiotic from the extracellular space, rather than the secretion of an extracellular enzyme *per se*, and that both periplasmic and extracellular β-lactamase activity can contribute to this social effect.

In Gram-positive bacteria, β-lactamases are generally secreted into the extracellular environment where they can break down β-lactam antibiotics outside the cell wall [37]. BlaZ, the best-studied β-lactamase of *Staphylococcus aureus*, illustrates this: BlaZ is produced in the cytoplasm and then exported so that a substantial fraction of the enzyme is present outside the cell, where it can inactivate penicillin-class antibiotics before they reach their targets [37]. *Staphylococcus aureus* also exports active BlaZ in membrane vesicles, and these vesicles can protect neighbouring, susceptible bacteria from β-lactams [42]. Because there is no outer membrane in Gram-positive bacteria, secreted β-lactamase can diffuse more freely into the environment and is more likely to act as a shared resource, providing a natural route to cooperative resistance [43]. A key question for future work is the relative importance of social effects in Gram-positive bacterial species relative to Gram-negative species.

### Anchoring

Bacterial cells have evolved structural adaptations to limit the ability of their neighbours to exploit their public good production. Bruce et al*.* showed that tiny hair-like structures, known as fibrils, were effective at limiting the ability of cheats to invade *in vitro* [44]. Anchoring is often regarded as a method of privatisation: by keeping a molecule close to or attached to the producing cell, the benefits of its activity accrue mainly to that cell rather than to neighbours. Siderophores, for example, can be retained to ensure that sequestered iron is preferentially taken up by the siderophore-producing individual [45]. β-lactamases can also be physically anchored to bacterial membranes: Capodimonte et al*.* used bioinformatic tools to predict that around 60% of OXA class D β-lactamases (that hydrolyse penicillins, including oxacillin) are lipidated and anchored in *Acinetobacter* spp., and experimentally showed that two clinically significant carbapenemases were membrane-bound [39].

In Gram-negative bacteria, anchoring can also influence how efficiently β-lactamases are incorporated into outer-membrane vesicles. Capodimonte et al*.* highlighted that membrane anchoring of OXA β-lactamases in *Acinetobacter* favours their extracellular secretion in vesicles and therefore enhances their ability to provide cooperative resistance [39]. Gonzalez et al*.* provided further insight into β-lactamase lipidation and anchoring, showing that specific lipidation motifs promote outer-membrane association and packaging into vesicles [46]. Recent work has confirmed that lipidated OXA β-lactamases are selectively enriched in outer-membrane vesicles and that mutating the lipidation site dramatically reduces vesicle-mediated export, providing a clear mechanistic link between anchoring, vesicle loading, and extracellular enzyme activity [39]. In this way, membrane anchoring in Gram-negative bacteria can enhance social effects, by biasing β-lactamase towards vesicle-mediated export and increasing the extent to which susceptible neighbours share in the benefits of resistance.

In Gram-positive bacteria, there is no outer membrane, so these species potentially rely more on anchoring to prevent enzymes from diffusing away from the cell surface [12,47]. Membrane anchoring in Gram-positive bacteria may help to privatise resistance and reduce opportunities for exploitation by non-producing cheats. Thus, the same molecular mechanism—lipidation and anchoring—may have opposite social consequences in Gram-negative and Gram-positive bacteria, depending on whether it promotes export in outer-membrane vesicles or restricts enzyme to the producing cell (Figure 3).

## Counter-resistance and clinical applications

Not all β-lactam antibiotics are equally vulnerable to β-lactamase enzymes, and clinical practice increasingly relies on β-lactam/β-lactamase inhibitor combinations to evade common enzymes [48]. These strategies are usually conceived at the level of individual cells, but as we have discussed, cooperative resistance means that β-lactamase production can protect neighbouring susceptible bacteria as well as producers. This distinction matters because inhibitors or dosing regimens that reduce extracellular activity will have a relatively strong impact on the social component of resistance.

Recognising that β-lactamase-mediated resistance can be social as well as private has several implications for therapy design [49]. Firstly, it suggests that treatment strategies should be evaluated not only for their impact on minimal inhibitory concentrations in pure cultures, but also for how they alter selection on producers versus non-producers in mixed communities, including the possibility that some inhibitor combinations increase the relative frequency of resistant strains. Secondly, it highlights the potential of interventions that specifically target the social component of resistance—for example, by disrupting outer-membrane vesicle production or activity—alongside broader proposals to develop ‘evolution-resistant’ anti-virulence therapies that act on cooperative traits [50,51]. More generally, identifying when β-lactamase behaves as a social trait, and when it does not, could improve predictions about which resistance mechanisms are most likely to spread under different treatment regimens and help to prioritise interventions that are less vulnerable to being undermined by social evolution.

## Conclusion

The present review highlights reasons why the protective effects of β-lactamase enzymes may vary. Subcellular localisation, membrane anchoring, outer-membrane vesicle-mediated export, and population structure all influence whether β-lactamase mainly benefits the producing cell or also protects non-producers. Experimental and genomic studies indicate that β-lactamase-mediated resistance can behave as a public good under some conditions, is subject to kin selection in natural populations, and can generate classic social dilemmas with cooperators and cheats. Taken together, these observations imply that β-lactamase-mediated resistance spans a continuum from effectively private to highly cooperative, and that social interactions between bacterial cells are an integral part of how β-lactamase evolves and influences the success or failure of antibiotic therapy.

## Summary

- β-lactamase-mediated resistance sits on a continuum from more private to more cooperative depending on enzyme identity, localisation, export, and population structure.
- Mechanistic details—periplasmic retention, membrane anchoring, and packaging into outer-membrane vesicles—shape how far β-lactamase activity extends beyond the producing cell and potentially influences how ‘social’ resistance becomes.
- Experimental work shows that different β-lactamases and ecological conditions generate variation in cross-protection of susceptible neighbours.
- Population-genomic analyses indicate that β-lactamase genes share signatures with other cooperative traits, with patterns of polymorphism and divergence consistent with kin selection acting on social resistance in natural bacterial populations.
- Recognising when β-lactamase behaves as a social trait has implications for therapy design, including how β-lactam/β-lactamase inhibitor combinations shape selection on producers versus non-producers and how interventions might target the social component of resistance.

## Open Access

Open access for this article was enabled by the participation of the University of Oxford in an all-inclusive Read & Publish agreement with Portland Press and the Biochemical Society under an agreement with JISC.
